# How choosy should I be? The relative searching time predicts evolution of choosiness under direct sexual selection

**DOI:** 10.1098/rspb.2014.0190

**Published:** 2014-06-22

**Authors:** Loïc Etienne, François Rousset, Bernard Godelle, Alexandre Courtiol

**Affiliations:** 1 Institut des Sciences de l’Évolution, Université Montpellier II, CNRS, Montpellier 34095, France; 2 Leibniz Institute for Zoo and Wildlife Research, Berlin 10315, Germany

**Keywords:** choosiness, direct benefits, mate choice, operational sex ratio, relative searching time, sexual selection

## Abstract

Most theoretical research in sexual selection has focused on indirect selection. However, empirical studies have not strongly supported indirect selection. A well-established finding is that direct benefits and costs exert a strong influence on the evolution of mate choice. We present an analytical model in which unilateral mate choice evolves solely by direct sexual selection on choosiness. We show this is sufficient to generate the evolution of all possible levels of choosiness, because of the fundamental trade-off between mating rate and mating benefits. We further identify the relative searching time (RST, i.e. the proportion of lifetime devoted to searching for mates) as a predictor of the effect of any variable affecting the mating rate on the evolution of choosiness. We show that the RST: (i) allows one to make predictions about the evolution of choosiness across a wide variety of mating systems; (ii) encompasses all alternative variables proposed thus far to explain the evolution of choosiness by direct sexual selection; and (iii) can be empirically used to infer qualitative differences in choosiness.

## 1. Introduction

Understanding the evolution of mate choice remains a theoretical challenge [1,2] despite much empirical support for its adaptive significance [3,4]. This discrepancy may have emerged because most theoretical works have focused on complex scenarios, whereas the analysis of common and simple mechanisms has attracted little interest among theoreticians [5].

In particular, most models have studied the evolution of female choice by sexual selection when selection favours a male's ornament and/or quality, but not directly the genes responsible for mate choice. Famous examples of such indirect selection models are the Fisher–Lande–Kirkpatrick model [6–8] and the so-called good-genes models [9–12]. These models imply the existence of benefits that enhance the reproductive success not of the choosy individuals themselves, but of their offspring. However, only a few empirical studies have identified such indirect benefits [13–18]. In addition, attempts have been made to estimate the strength of indirect selection in natural populations [19–21], and they find no significant evidence for its impact on the evolution of mate choice [22]. By contrast, it is well established that mate choice is directly selected in a wide variety of organisms [3,23]. Moreover, this direct selection may exert a greater influence on the evolution of choice than indirect selection [24–26]. Direct selection originates from direct benefits such as increased fertility, parental care, protection, territory, food, nuptial gifts or risk reduction (for a review see [3], ch. 8).

Direct benefits imply that choice can be favoured by sexual selection (defined as the differences in reproductive success arising as a result of both the number of matings and the quality of mates). Nonetheless, direct benefits may be counteracted by various costs of mate choice, and this may explain why the intensity of choice (i.e. choosiness) varies widely both between and within species [27]. Examples for costs of choosiness include: the increased predation risk caused by mate searching [11,28], the risk of being injured [29] or eaten by mates [30] and the risks inherent to fighting with same-sex conspecifics [31–33]. These costs may affect the survival of choosy individuals, and different levels of choosiness can evolve according to the respective intensities of these costs and direct benefits. However, even when direct benefits are present and costs on survival are absent, a maximal level of choosiness may not be selected, because choosiness is already associated with an unescapable cost. Indeed, the time spent searching for mates increases systematically with choosiness, and thus reduces the mating rate, because the choosier an individual is, the rarer are the individuals qualifying as mates [34]. Moreover, this temporal cost may be enhanced by the fact that high-quality mates have already mated with other choosers and are thus unavailable for some time.

That choosiness is intrinsically associated with an increase in mating benefits and a decrease in mating rate implies a trade-off between these two fitness components [29,30,35–37]. Here, we study the influence of this trade-off, by building an analytical model in which choosiness evolves only by direct sexual selection, contrary to previous studies that have also included other selective pressures [28–30,36]. The trade-off must operate in most cases of mate choice, because it only requires that: (i) mates vary with respect to the benefits they can supply, and (ii) the mating rate varies according to the level of choosiness. We choose to explore the impact of general features influencing the trade-off (the intersexual encounter rate, the length of latency after mating, the lifetime and the distribution of qualities among mates) in a simple behavioural context where choice is unilateral (i.e. only one sex can be choosy) without condition dependence. This allows us to describe the evolution of choosiness in a wide range of situations. As such, our approach complements the study by Johnstone *et al.* [38], who focused on mating patterns emerging from mutual condition-dependent mate choice and generalizes models that have assumed either males to be always available for mating [39], or individuals to mate only once [40–45]. Our study reveals the full range of choosiness that direct sexual selection is able to generate. This extends approaches that have studied the evolution of choosiness without investigating the values that choosiness can attain at the evolutionary equilibrium, either by distinguishing only between choosy and non-choosy individuals [46,47], or by calculating the optimal choosiness without considering that it must be constrained by the choosiness of other same-sex individuals [35].

Here, we show that direct sexual selection is sufficient to generate the evolution of all possible levels of choosiness. We further find that under direct sexual selection, the evolution of choosiness can be predicted from the relative searching time (RST, i.e. the proportion of lifetime devoted to searching for mates), which is a more general predictor than previously proposed ones.

## 2. The model

### (a) The life cycle

We build a discrete time model of an infinite population at demographic equilibrium. We consider two sexes with a sex ratio of 1 : 1. For both sexes, the lifetime of individuals is set by the probability *s* of surviving from one time step to the next, which is identical for all individuals and constant across their entire life. The average lifetime is thus 1/(1 − *s*) (the time step during which an individual dies is included in lifetime). At each time step, each individual randomly encounters an opposite-sex individual with probability *e*. We assume that each individual can only mate with one individual per time step, but mating several times over the lifetime is possible.

Our model corresponds to a situation of unilateral choice in which females choose males according to their quality but males will willingly mate with any female. Each male is characterized by a value of quality *q* between 0 and 1, which is strictly environmentally determined. This prevents the emergence of linkage disequilibrium, and thus indirect selection, in our model. The distribution of male quality is constant across generations. We allow this distribution to take any form, but the following calculations will illustrate the case of a uniform distribution (see the electronic supplementary material for the general case). Each female is characterized by a level of choosiness *ϕ*, which represents the minimal male quality the female will accept. We assume that this threshold is entirely genetically determined by a single locus, for which there are an infinite number of possible alleles (any real number between 0 and 1). We also assume that females make no error in assessing the quality of males, so that a female with choosiness *ϕ* only mates with males with quality *q* ≥ *ϕ*.

After mating, paired individuals enter a latency period (also referred to as ‘time out’ period in some papers: e.g. [46]) and become unavailable for mating. Biologically, latency can result from parental care, gamete depletion, mate guarding or any other state that prevents individuals from remating instantly. The length of this period can be expressed through a probability of entering or remaining in latency, denoted *l*. This formalism eases comparisons between the effects of the different parameters and leads to results qualitatively similar to those obtained when a fixed duration of latency is modelled. We allow *l* to differ between females (*l*_♀_) and males (*l*_♂_). Moreover, we assume that the durations of latency are independent between the female and the male of a given mating pair (but see the electronic supplementary material for modelling the opposite case). At the end of this latency period, individuals become available for mating again.

**Figure 1.**
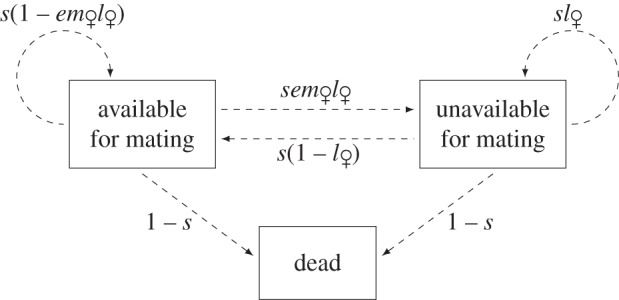
The life cycle of a female. At each time step: *s* is the probability that she survives, *e* the probability that she encounters a male, *m*_♀_ the probability that she mates with this male and *l*_♀_ the probability that she engages or remains in latency.

A female switches from being available for mating to unavailable upon meeting all of the following conditions: (i) she does not die (with probability *s*), (ii) she encounters a male (*e*), (iii) this male is available and of sufficient quality for mating (with this probability denoted as *m*_♀_, which is a function of the other parameters), and (iv) she enters into latency (*l*_♀_). Thus, the transition probability from the available state to the unavailable state between two time steps equals (*sem_♀_ l*_♀_). An unavailable female remains unavailable with probability 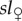, i.e. when she does not die (*s*) and remains in latency (*l*_♀_). Alternatively, an unavailable female becomes available again with probability *s*(1−*l*_♀_). Finally, there are two possibilities for an available female to remain in this state: either she mates but does not engage in latency (*sem*_♀_(1−*l*_♀_)), or she does not mate because of failing to encounter a potential mate or because the male is unavailable or of too low quality 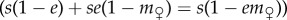. Hence, the transition probabilities between the states of the female life cycle are given in figure 1, and are summarized in the following matrix:2.1
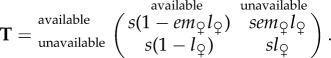

### (b) Calculating mating probability

We now describe the relationship between the mating probability of a focal female 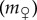 and the other parameters. First, this mating probability depends on the probability that the focal female finds the male she encounters to be acceptable, thus on her choosiness (*ϕ*). Second, 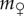 also depends on the availability of males, which in turn depends on the choosiness of other females in the population. Indeed, a male who is encountered can be in latency after a previous mating and thus unavailable for a new mating. To take this competition for mates into account, we use the framework of mutants and residents [48], assuming that all females in the population (i.e. residents) show the same level of choosiness (*ϕ*_p_) except for a focal female (i.e. the mutant), whose choosiness is *ϕ*. Two cases need to be considered. If the mutant female is choosier than residents (*ϕ* ≥ *ϕ*_p_), the males of sufficient quality to be chosen (*q* ≥ *ϕ*) may have previously mated with a resident female and still be in latency. We denote the probability for such a male to be available as *a*_♂_. If the choosiness of the mutant is lower than the resident one (*ϕ* < *ϕ*_p_), then males whose quality ranges from *ϕ* to *ϕ*_p_ are never chosen by resident females and are always available for mating with the mutant female. Males with quality higher than resident choosiness (*q* ≥ *ϕ*_p_) are available with probability *a*_♂_. Thus, in the case of a uniform distribution of male quality, the probability of mating for a mutant female with choosiness *ϕ* is2.2
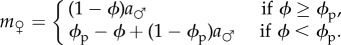


Using the property that the life cycle forms a Markov chain for which death is an absorbing state, we obtain from the matrix in equation (2.1) (see the electronic supplementary material)2.3
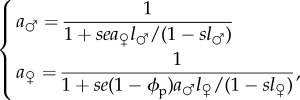
where 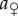 is the availability of resident females. Indeed, both the male and the female must be available to form a mating pair, thus 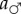 and 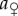 are necessarily related. By solving this system, we obtain the analytical expression for 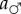 (see the electronic supplementary material).

### (c) Calculating fecundity

We consider lifetime fecundity of females, i.e. the number of offspring produced over all mating events. We assume that mating with a male *i* with quality *q_i_* is associated with a direct benefit *b_i_* = *q_i_* in terms of female reproductive success. We also consider that the number of offspring obtained from any mating event depends neither on the number of previous matings nor on the number of offspring obtained from these previous matings. Hence, the expected fecundity *F*(*ϕ*, *ϕ*_p_) of a female of choosiness *ϕ* in a resident population of choosiness *ϕ*_p_ is the product of her expected mating rate *r*(*ϕ*, *ϕ*_p_), her expected benefit per mating *b*(*ϕ*, *ϕ*_p_) and her expected lifetime (see the electronic supplementary material):2.4
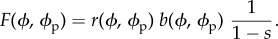


The expected mating rate *r*(*ϕ*, *ϕ*_p_) equals the probability that at a given time step the mutant female is available for mating 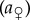, multiplied by the probability that she finds a male and mates with him at this time step 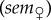. In the case of a mutant of choosiness equal to or higher than resident choosiness and a uniform distribution of male quality, we obtain from equations (2.2) and (2.4) (see the electronic supplementary material for the general case)2.5
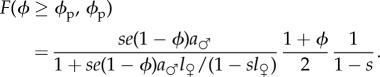


### (d) The evolution of choosiness and the trade-off

We have found that choosiness always evolves until it reaches an evolutionarily stable strategy (ESS), denoted ϕ*, regardless of the values of the parameters and of the distribution of mate quality (see the electronic supplementary material). This means polymorphism is never selected in our model. The derivative of mutant fecundity with respect to choosiness is null at ESS. Because fecundity is the product of the mating rate, the mating benefits and the lifetime (see equation (2.4)), and lifetime is not affected by choosiness, the ESS is attained when the relative increase *B* (B* at ESS) in the mating benefits equals the relative decrease *R* (R* at ESS) in the mating rate in absolute value, i.e. when2.6
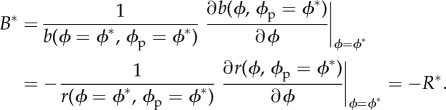
This means the evolution of choosiness depends on the form of the trade-off between the mating rate and mating benefits (electronic supplementary material, figure S1).

**Figure 2.**
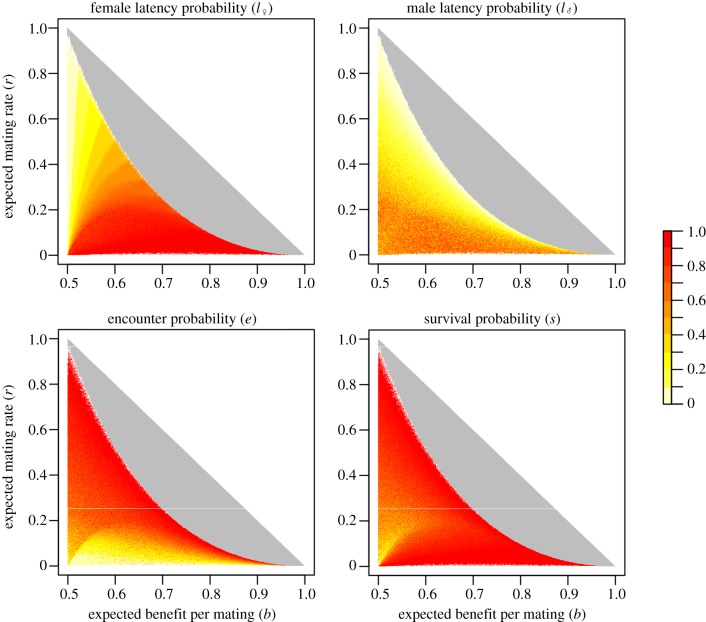
The mean value of each parameter for the different values of the trade-off between mating rate and mating benefits at ESS. The mating rate *r* is plotted against the mating benefits *b*. The space below the diagonal (grey+coloured area) corresponds to the possible combinations of *r* and *b*, and the coloured area represents evolutionarily stable cases. The colour scale indicates the mean value of each parameter for all possible combinations of *r* and *b* at ESS, because the same combination can be attained by different sets of parameters. These figures have been obtained by calculating *r* and *b* in 10^8^ cases that explore the entire range of the parameters (i.e. *e*, *s*, *l*_♀_ and *l*_♂_ varying between 0 and 1 whereas *ϕ* = *ϕ*_p_ = ϕ*). The quality of males follows a uniform distribution. (Online version in colour.)

We have calculated all the combinations of mating rate and mating benefits that result from all possible ESSs of choosiness and have found that our model allows for very different combinations of these fecundity components to evolve (figure 2). The distribution of these combinations depends on the parameters of our model (*e*, *s*, 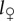 and 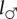) and on the distribution of mate quality. Some combinations correspond to a restricted set of parameter values, whereas others can occur over a much wider range of situations. For instance, in the case of a uniform distribution of mate quality, ESSs in which females have a high mating rate but can mate with low-quality males are possible only if encounter and survival rates are high while latency rates are low for both sexes. By contrast, ESSs in which females have a low mating rate but mate only with high-quality males are possible for all values of encounter and male latency rates, provided that the survival and female latency rates are high. Importantly, any level of choosiness can be an ESS in our model, even if we consider only one distribution of mate quality (e.g. uniform).

### (e) The evolution of choosiness and the relative searching time

We have found that the effect on the evolution of choosiness of any biological or ecological variable *z* affecting the mating rate *r* but not the mating benefits *b* is related to its effect on the relative amount of lifetime spent searching for mates, which we call the RST. Because we have assumed choosiness to be constant throughout lifetime, the RST can also be defined as the proportion of time of one reproduction event which is devoted to searching for mates. Using the ‘time in–time out’ terminology (which refers to the time spent, respectively, in the states ‘available for mating’ and ‘unavailable for mating’: e.g. [46]), the RST would be written as the ratio ‘time in’/(‘time in’ + 'time out’).

This result rests on two computation steps that we describe briefly here (see the electronic supplementary material for details). First, *z* affects the relative decrease *R* in the mating rate, but not the relative increase *B* in the mating benefits. Because the ESS is reached when B* =− R* (see equation (2.6)), the effect of *z* on the evolution of choosiness may therefore be deduced from its effect on R*. Indeed, we find that the change dϕ*/d*z* in choosiness at ESS caused by the variation of *z* has the same sign as the change in the relative decrease in the mating rate at ESS (R*) with *z*. This change is formally defined as a partial derivative ∂R*/*∂z*. In particular, although ϕ* is a function of *z* and appears in the expression of R* (see equation (2.6)), ϕ* is considered independent of *z* in this partial derivative. Thus, ∂R*/*∂z* does not include variation owing to the evolution of choosiness and therefore represents the *sensitivity* [49] of R* with respect to *z*. Second, at ESS the mating rate *r* is the inverse of the mean duration of one reproductive event. With some calculations, we find from this property that the sensitivity of the RST at ESS 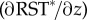 has the opposite sign of the sensitivity of the relative decrease in the mating rate at ESS (∂R*/*∂z*). Hence, the change in choosiness has the opposite sign of the sensitivity of the RST:2.7
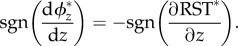


This result rests on the following assumptions: (i) *z* does not affect the distribution of mate quality, regardless of the form of the latter; (ii) choosiness does not affect survival; and (iii) choosiness does not affect the time spent in one latency period. This result may therefore be extended to any system of mate choice satisfying these assumptions.

**Figure 3.**
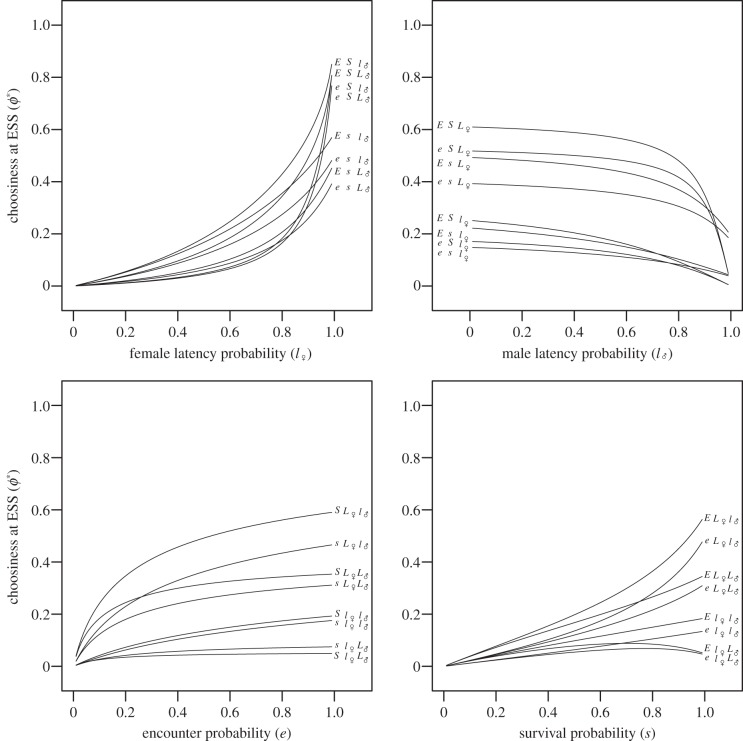
The effect of parameters on choosiness at ESS. The value of choosiness at ESS ϕ* (*y-*axis) is plotted against each parameter (*x-*axis). For each graph, the three other parameters vary between two values: 0.5/0.9 for *e*, *l*_♀_ and *l*_♂_ and 0.9/0.999 for *s*. Parameters set to the higher value are written in capital letters.

Because all our model parameters affect the mating rate but not the mating benefits, we can use the sensitivity of the RST to predict their effect on choosiness at ESS (figure 3). When female latency probability 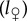 increases, the time females spend in latency increases, which decreases the RST because the lifetime is constant. Therefore, female latency selects for choosiness. When male latency probability 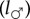 increases, the time females spend before encountering a male who is available increases, which increases the RST. Therefore, male latency reduces the choosiness at ESS. When encounter probability (*e*) increases, the time females spend before encountering a potential mate decreases, which decreases the RST. This implies that a higher encounter rate selects for greater choosiness. Finally, when survival probability (*s*) increases, the proportion of lifetime spent in latency increases with *s* in both sexes. This is because when death occurs, the individual is always replaced by an available one, whether the dead one was in latency or not. Then, when the time spent in latency increases in one sex, the time spent searching for mates increases for individuals in the other sex. The resulting effect on the RST depends on the values assigned to the latency parameters. For instance, if male latency probability is much lower than female latency probability, the increase with *s* in the time spent in latency outweighs the increase in the time spent searching for mates in females, leading to a decrease in female RST. Thus an increase in the survival probability selects for increased choosiness in that case. The opposite result is obtained if male latency probability is much higher than female latency probability.

**Figure 4.**
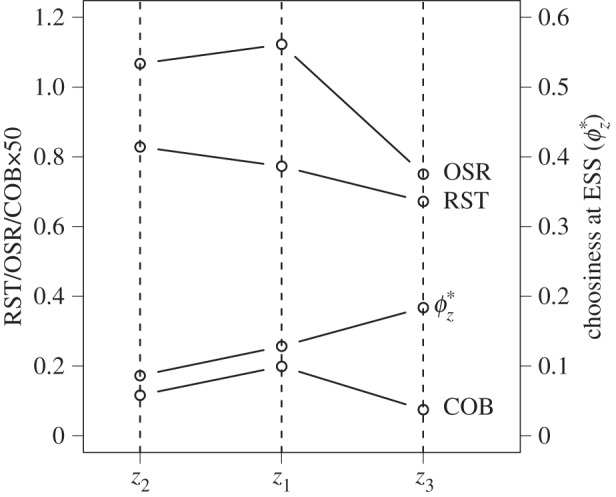
Comparison of the predictive power of the RST, the OSR and the COB. The values of the RST, the OSR, the COB and choosiness at ESS are plotted for three different parameter settings (*z*_1_: *e* = 0.1, *l*_♀_ = 0.8 and *l*_♂_ = 0.7; *z*_2_: *e* = 0.1, *l*_♀_ = 0.7 and *l*_♂_ = 0.6; *z*_3_: *e* = 0.9, *l*_♀_ = 0.6 and *l*_♂_ = 0.7; *s* equals 0.999 and the distribution of quality is uniform in all cases). During the change *z*_1_ to *z*_2_, the three metrics correctly predict the variation of choosiness at ESS, while during the change *z*_1_ to *z*_3_, only the RST yields correct predictions. Note that when computing the RST, the value of choosiness remains fixed to 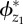, in order to represent the sensitivity of the RST.

Previous models have proposed other predictors for the evolution of choosiness under direct sexual selection, including the time invested in breeding [37,38], the adult sex ratio [50], the operational sex ratio (OSR, [51]) and the cost of breeding (COB, [46,47]). Under our formalism, the effects of the two former predictors can be entirely captured by, respectively, considering a change in *l* and *e*, while the two latter predictors can be, respectively, written as 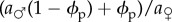 and 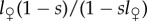 (see the electronic supplementary material). This means that for a fixed value of choosiness at ESS, they all affect the mating rate but not the mating benefits and are thus encompassed by the sensitivity of the RST. In particular, the OSR and the COB have both been proposed to be positively correlated with choosiness at ESS. Any change in the OSR or the COB explaining a change in choosiness at ESS will be correctly captured by a change in the RST (e.g. when latency decreases for both sexes, as during the change *z*_1_ to *z*_2_ in figure 4). However, the RST can also vary and thus predict correctly the evolution of choosiness, while the OSR and the COB remain constant or change in the opposite way as choosiness (e.g. when the encounter rate increases while female latency decreases, as during the change *z*_1_ to *z*_3_ in figure 4).

## 3. Discussion

We have modelled the evolution of mate choice in a very simple case: mate choice is (i) unilateral, (ii) based on one cue of quality that is directly accessible, (iii) expressed as a fixed threshold with no condition dependence, (iv) provides direct benefits alone and thus only evolves by direct selection, and (v) does not affect survival and thus only evolves by sexual selection. We have found that despite this simplicity, the model is sufficient to generate the evolution of all possible levels of choosiness. This is because the form of the trade-off between mating rate and mating benefits varies greatly according to the biological context (here described by the encounter rate, the lifetime, the time spent in latency and the distribution of quality among mates). To our knowledge, our model is the first to compute the possible levels of choosiness one can observe at the equilibrium when only direct sexual selection operates. As such, we supplement previous studies that have qualitatively explored the evolutionary effect of this trade-off [38,39,41]. In addition, we have identified a predictor for the evolution of choosiness, the sensitivity of the RST, which encompasses all previously proposed metrics.

Our model predicts the existence of an ESS for choosiness, regardless of the values of the four parameters of the model and of the distribution of mate quality. Hence, we extend the results of Gowaty & Hubbell [39] who also found an ESS for choosiness but in the particular case of the absence of competition for mates (by assuming a null latency for males). By contrast, in our model some competition emerges as the result of the unavailability of certain mates. This competition is similar to scramble competition in community ecology, which results from the consumption of a resource (here, the mates) by other competitors (here, the other conspecific choosers) without physical interference between the competitors. The importance of scramble competition in sexual selection has been demonstrated, but it has often been considered in the non-choosing sex (e.g. [52–54]), while here we focus on the competition between choosers. This kind of competition is known to reduce choosiness in several species (e.g. [55–57]), which may explain why even in the absence of survival costs on choosiness, low levels of choosiness can evolve. Moreover, combined with condition-dependent expression of choosiness [58], this may select for plasticity or polymorphism in choosiness [31–33,38,41].

We have shown that the evolution of choosiness can be predicted from the RST (i.e. the proportion of lifetime devoted to searching for mates). Formally, we have found that the effect on choosiness at the ESS of any variable affecting the mating rate without affecting the mating benefits (regardless of the distribution of the latter) is opposite to the sensitivity of the RST with respect to this variable, i.e. the variation of the RST is directly attributable to a small increase in this variable. Importantly, the sensitivity of the RST does not include variation of the RST caused by a change in the value of choosiness. We have found that this result can be applied to any system of mate choice in which choosiness does not affect (i) survival, and (ii) the time spent in one latency period. This may reflect many different biological situations. We have assumed that only females choose their mates, but our model can also effectively describe cases of male choice. Indeed, the only difference between the sexes concerns the length of latency, which is encoded in an independent parameter for each sex. Replacing male and female parameters in the equations of the model is therefore sufficient for switching from female choice to male choice. Moreover, depending on the relative lengths of latency period and lifetime, our model is able to represent the entire range of mating rates existing in nature, from very low (e.g. because of a very low encounter rate, such as in redback spiders [59]) to very high (e.g. because of a very short latency period, such as in stalk-eyed flies [60]).

We have found that previously proposed predictors such as the time invested in breeding [37,38], the adult sex ratio [50], the OSR [51] and the COB [46,47] are all encompassed by the RST. This means that any prediction about the evolution of choosiness made with one of these variables can be deduced from the sensitivity of the RST. Conversely, we have identified cases in which a variation of the RST correctly predicts the change in choosiness at ESS while other predictors remain constant or yield opposite predictions. The failure of these other predictors is known empirically: e.g. a male-biased OSR can be associated with male choosiness higher than female choosiness in several species [61–63]. We argue that in such situations, the RST will be able to predict correctly the variation of choosiness, provided that the conditions upon which the RST rests are satisfied. In particular, the RST may be no longer sufficient if choosiness significantly affects survival. Nonetheless, the aforementioned predictors have to be used under the same restrictive conditions as for the RST. This means that when choosiness evolves solely by direct sexual selection, the sensitivity of the RST is the most general predictor among those that have been proposed thus far.

Computing the sensitivity of the RST allows one to make predictions about the effects of our parameters on the evolution of choosiness. If the sensitivity of the RST with respect to a given parameter is positive, an increase in the value of this parameter will decrease the level of choosiness at ESS, and *vice versa*. Focusing on the sign of the sensitivity of the RST often makes predictions more intuitive than if the RST was ignored. For instance, it makes sense that an increase in the encounter rate or the latency of choosers leads to a decrease in the RST. This explains why these two parameters act positively on the value of choosiness at ESS, which is empirically attested (choosiness increases with encounter rate [64–66], and decreases with reproduction rate, i.e. increases with latency [67,68]). The similar effect of these two parameters had been already shown by previous models [28,35,38,39,41,46,47], and the RST provides a unified explanation for these results.

Beyond the effect of these parameters, measuring the sensitivity of the RST in nature can be used to predict the effect of more complex biological or ecological variables on choosiness. Let us consider the impact of density, in the particular case of mating systems with nuptial prey gifts given by males to females, which is common in insects [69]. In this context, an increase in density can select for female choosiness by increasing the encounter rate, as it has been shown in a scorpionfly [70]. However, the opposite effect has been observed in Mormon crickets [71]: density was negatively correlated with choosiness, because it increases food competition between males and thus the time necessary to find prey, which corresponds to an increase in male latency. This example shows that the effect of the target variable on choosiness is not always trivial to predict. In such cases, measuring the sensitivity of the RST with respect to this variable allows us to obtain predictions. The RST may be empirically accessible by measuring the values of encounter, latency and survival rates in the wild. However, measuring precisely these four parameters at the same time may be difficult in many cases. Alternatively, one can use any proxy that could give an estimation of the RST (e.g. the time spent sampling mates or courting divided by the total time spent in mate search and in latency). Then, this proxy has to be measured before and after the variable considered has changed (naturally or during the course of an experiment). Importantly, the first measurement has to be done once choosiness has reached an evolutionary equilibrium, and the second before choosiness changes (because of selection or phenotypic plasticity). In this case only, the difference between the two estimations of the RST corresponds to the sensitivity of the RST. Therefore, to correctly predict changes in choosiness, experiments or observations have to be realized on a short time-scale and in stable environmental conditions with the exception of the variable one wants to study.

Because direct sexual selection has the potential to generate all possible levels of choosiness, we encourage theoreticians to consider it as a crucial process determining the evolution of mate choice. Nevertheless, this does not mean that direct sexual selection is the only force acting on choosiness. Other selective pressures influencing the evolution of mate choice may play an important role, such as indirect selection [6,7,9–12], natural selection on choosiness [11,29–33] or antagonistic selection between the sexes [72,73]. All these processes can be described in terms of genetic variances, covariances and selection gradients within a quantitative genetic formalism [1,2]. Because direct sexual selection alone is considered in our model, within such a formalism all these variables are assumed to be null except the variance of choosiness and the sexual selection gradient on choosiness. Thus, our model probably represents the simplest model of sexual selection of mate choice one can conceive. We therefore suggest that the aforementioned mechanisms should be studied in addition to direct sexual selection as modifications of our basic model, rather than in isolation.
